# Traditional Chinese medicine xin-mai-jia recouples endothelial nitric oxide synthase to prevent atherosclerosis *in vivo*

**DOI:** 10.1038/srep43508

**Published:** 2017-03-02

**Authors:** Ya-Ling Yin, Mo-Li Zhu, Jia Wan, Chong Zhang, Guo-Pin Pan, Jun-Xiu Lu, Song Ping, Yuan Chen, Fan-Rong Zhao, Hai-Ya Yu, Tao Guo, Xu Jian, Li-Ying Liu, Jia-Ning Zhang, Guang-Rui Wan, Shuang-Xi Wang, Peng Li

**Affiliations:** 1 College of Pharmacy and School of Basic Medical Sciences, Xinxiang Medical University, Xinxiang, 453003, China; 2 Department of Drug and Cosmetics Supervision, Henan Food and Drug Administration, Zhengzhou, 450018, China; 3 The Key Laboratory of Cardiovascular Remodeling and Function Research, Qilu Hospital, Shandong University, Jinan, 250012, China; 4 Sanquan College of Xinxiang Medical University, Xinxiang, 453003, China; 5 Department of Neurology, The People’s Hospital of Xishui County, Huangang, Hubei, China; 6 Department of Pharmacology, Xiangya School of Pharmaceutical Sciences, Central South University, Changsha, China; 7 Biology and Chemistry, Denison University, Granville, OH, USA

## Abstract

Endothelial dysfunction, which is caused by endothelial nitric oxide synthase (eNOS) uncoupling, is an initial step in atherosclerosis. This study was designed to explore whether Chinese medicine xin-mai-jia (XMJ) recouples eNOS to exert anti-atherosclerotic effects. Pretreatment of XMJ (25, 50, 100 μg/ml) for 30 minutes concentration-dependently activated eNOS, improved cell viabilities, increased NO generations, and reduced ROS productions in human umbilical vein endothelial cells incubated with H_2_O_2_ for 2 hours, accompanied with restoration of BH4. Importantly, these protective effects produced by XMJ were abolished by eNOS inhibitor L-NAME or specific eNOS siRNA in H_2_O_2_-treated cells. In *ex vivo* experiments, exposure of isolated aortic rings from rats to H_2_O_2_ for 6 hours dramatically impaired acetylcholine-induced vasorelaxation, reduced NO levels and increased ROS productions, which were ablated by XMJ in concentration-dependent manner. *In vivo* analysis indicated that administration of XMJ (0.6, 2.0, 6.0 g/kg/d) for 12 weeks remarkably recoupled eNOS and reduced the size of carotid atherosclerotic plaque in rats feeding with high fat diet plus balloon injury. In conclusion, XMJ recouples eNOS to prevent the growth of atherosclerosis in rats. Clinically, XMJ is potentially considered as a medicine to treat patients with atherosclerosis.

Atherosclerosis is an arterial disease that involves chronic inflammation from initiation to progression and, eventually, plaque rupture, thrombosis, and occlusion of the vessel^1^,^2^. In the stage of atherosclerotic growth, the abnormal homeostatic functions of the endothelium are appeared, promoting an inflammatory response. The most important factor for the maintenance of vascular homeostasis is nitric oxide (NO), derived from L-arginine in the catalysis of endothelial NO synthase (eNOS).

A critical determinant of eNOS function is the availability of tetrahydrobiopterin (BH4)^3^. Recent studies^4^,^5^ indicate that eNOS must be fully saturated with BH4 to couple NADPH oxidation to NO production. Under BH4 deficiency, eNOS functions in an “uncoupled” state in which NAD(P)H-derived electrons are added to molecular oxygen, rather than L-arginine, leading to the productions of reactive oxygen species (ROS)^6^. Uncoupled eNOS has been implicated in a number of vascular diseases, such as hypertension^7^, atherosclerosis^8^, and diabetes^4^. BH4 supplementation in vessel rings from animals with atherosclerosis, diabetes, or hypertension prevents endothelial dysfunction^9^,^10^,^11^.

Xin-mai-jia (XMJ) is a traditional Chinese compound prescription and has been approved by the State Food and Drug Administration of China in 2010. We have previous reported that XMJ decreases blood lipid levels and improves the functions of artery and heart by performing small scale clinical trial. However, whether and how XMJ functions as an anti-atherosclerotic drug is poorly understood.

Based on the aforementioned studies, we hypothesized that XMJ may recouple eNOS to suppress atherosclerotic plaque growth. In the present study, we reported that XMJ increased intracellular BH4 levels and NO releases, and reduced oxidative stress in cultured HUVECs. In rats, administration of XMJ prevented endothelial dysfunction and inhibited the progression of atherosclerosis.

## Materials and Methods

### Materials

XMJ crude drugs were purchased from Beijing Tong Ren Tang Co, China. Primary antibodies against eNOS, p-eNOS at ser1177, GTPCH1, and GAPDH, and secondary antibodies were obtained from Santa Cruz Biotechnology Company (Santa Cruz, CA). Lovastatin, NG-Nitro-L-arginine Methyl Ester (L-NAME), acetylcholine (Ach), phenylephrine (PE), and sodium nitroprusside (SNP) were obtained from Sigma (St. Louis, MO). Zhibituo (ZBT) was purchased from Chengdu DioJiu Hong Pharmaceutical Industry co, China. 4-Amino,5-aminomethyl-2′,7′-difluorescein (DAF), dihydroethidium (DHE), and 3-(4,5)-dimethylthiahiazo(-z-y1)-3,5-di- phenytetrazoliumromide (MTT) were from Cayman Chemical Company (Ann Arbor, Michigan, USA). Control and eNOS siRNAs (5′-UGUGUAUGGAUGAGUAUGACG-3′) were from Santa Cruz Biotechnology Inc. The siRNA delivery agent, Lipofectamine 2000, was from Invitrogen (Carlsbad, CA).

### Animals

Male Sprague-Dawley rats (8 ± 2 weeks old, 180 ± 20 g) were purchased from the Center of Experiment Animals, Xinxiang Medical University (Xinxiang, Henan, China). Rats were housed in temperature-controlled cages with a 12-hour light-dark cycle. This animal study was carried out in strict accordance with the recommendations in the Guide for the Care and Use of Laboratory Animals of the National Institutes of Health. The animal protocol was reviewed and approved by Xinxiang Medical University, Institute of Animal Care and Use Committee.

### Components, preparation, and chemical analysis of XMJ

XMJ is a Chinese medicinal formulation that is available in capsule form. The formula contains 10–35% functional red kojic rice powder, 1–10% kudzu flavonoid powder, 1–8% soybean isoflavone powder, 1–8% bamboo leaf flavone powder, 1–8% resveratrol powder, 1–6% hawthorn powder, 0.1–0.2% powdered hippocampus body, 0.008–0.04% astaxanthin powder, 0.1–0.3% menthol powder and 20–50% resistant starch. They were ground to superfine powder with the diameter of 10 μm or less by a micronizer and prepared as capsules. To reduce the dose variability of XMJ capsule among different batches, the species, origin, harvest time, medicinal parts, and concocted methods for each component were strictly standardized. Moreover, high performance liquid chromatography was applied to quantitate the components of the XMJ capsule. Five major components of the aqueous extract of XMJ capsule included 8-β-D-Glucopyranosyl-7-hydroxy-3- (4-hydroxyphenyl)-4H-1-benzopyran-4-one (3.79 mg/g), 4′,7-Dihydroxyisoflavone 7-Hydroxy-3-(4-hydroxy-phenyl)-chromone 7-Hydroxy-3-(4-hydroxy-phenyl)-4H-1-benzo-pyran-4-one (2.23 mg/g), 4-Hydroxybenzyl alcohol 4-O-bata-D-glucoside (1.88 mg/g). 5-(4-hydroxystyryl)-benzene-1,3-diol (1 mg/g), 3,3′-Dihydroxy-beta,beta-carotene-4,4′-dione (0.79 mg/g).

### Cell culture

HUVECs from America Type Collection Center (ATCC) were grown in endothelial cells basal medium (Clonetics Inc. Walkersville, MD) supplemented with 2% FBS, penicillin (100 U/ml), and streptomycin (100 μg/ml). In all experiments, cells were between passages 3 and 8. All cells were incubated at 37 °C in a humidified atmosphere of 5% CO_2_ and 95% air. Cells were grown to 70–80% confluency before being treated with different agents.

### Gene expression array

Agilent Whole Genome Oligo Microarrays (one-color) was used to do Gene expression array. Briefly, HUVECs were harvested and were subjected to RNA extortion by using TRIzol Reagent (Invitrogen life technologies). After preparation of labeling reaction, purified RNA was labeled and amplified RNA. Hybridization and microarray wash were performed to amplified RNA. After scanning the slides by using Agilent Microarray Scanner (Agilent p/n G2565BA), data were extracted using Agilent Feature Extraction Software and analyzed.

### Transfection of siRNA into cells

Transient transfection of siRNA was carried out according to Santa Cruz’s protocol^12^. Briefly, the siRNAs were dissolved in siRNA buffer (20 mM KCl; 6 mM HEPES, pH 7.5; 0.2 mM MgCl_2_) to prepare a 10 μM stock solution. Cells grown in 6 well plates were transfected with siRNA in transfection medium (Gibcol) containing liposomal transfection reagent (Lipofectamine RNAimax, Invitrogen). For each transfection, 100 μl transfection medium containing 6 μl siRNA stock solution was gently mixed with 100 μl transfection medium containing 6 μl transfection reagent. After a 30-min incubation at room temperature, siRNA-lipid complexes were added to the cells in 1.0 ml transfection medium, and cells were incubated with this mixture for 6 h at 37 °C. The transfection medium was then replaced with normal medium, and cells were cultured for 48 hours.

### Western blot analysis

Cells were lysed in cold RIPA buffer. Protein concentrations were determined with a bicinchoninic acid protein assay system (Pierce, Rockford, IL). Proteins were subjected to Western blots using ECL-Plus, as described previously^13^. The intensity (area X density) of the individual bands on Western blots was measured by densitometry (model GS-700, Imaging Densitometer; Bio-Rad). Distribution of eNOS dimer and monomer was assayed by performed western blot under 4 °C condition.

### eNOS activity assay

eNOS activity was monitored by L-[^3^H]citrulline production from L-[^3^H]arginine as described previously^14^. Briefly, protein samples were incubated in reaction buffer containing 1 mM L-arginine, 100 mM NADPH, 1 mM tetrahydrobiopterin, 0.2 μCi of L-[^3^H]arginine (>66 Ci/mmol), and Nω-hydroxy-nor-L-arginine (10 μM). The reaction was performed at 37 °C for 15 min and the mixture was separated by Dowex-50W ion-exchange chromatography in 20 mM HEPES (pH 5.5), 2 mM EDTA, and 2 mM EGTA, and the flow-through was used for liquid scintillation counting.

### Measurement of intracellular NO and ROS productions

For detections of intracellular NO and ROS, cells grown in 24-well plates were incubated for 30 min in the presence of 10 μM DAF or DHE in PBS in the dark at 37 °C. Cells were then washed with PBS to remove excessive DAF or DHE, and the change in fluorescence was recorded for 15 min at room temperature using a microplate reader (FL 600, Bio-Tek). The fluorescent intensity was recorded by fluorescent reader at the wave of excitation (485 nm) and emission (530 nm for DAF, 545 nm for DHE). Control was setup as 100%.

### Evaluation of cell viability

Cell viability was assayed by using MTT as described previously^15^. Cells were seed into 96-well plate at the density of 10000/ml and incubated for 24 hours. After treatment, 10 μl MTT (5 mg/ml) was added into cultured medium in each well for 2–4 hours until purple precipitate is visible. After removal of culture medium, 75 μl dimethyl sulphoxide was added to each well and leave the cells at room temperature in the dark for 2 hours. The absorbance at 570 nm was recorded.

### Organ chamber

Organ chamber study was performed as described previously^14^. Rats were sacrificed under anesthesia by intravenous injection with pentobarbital sodium (30 mg/kg). The descending aorta isolated by removing the adhering perivascular tissue carefully was cut into rings (3–4 mm in length). Aortic rings were suspended and mounted to organ chamber by using two stainless clips. The rings were placed in organ baths filled with Kreb’s buffer of the following compositions (in mM): NaCl, 118.3; KCl, 4.7; MgSO_4_, 0.6; KH_2_PO_4_, 1.2; CaCl_2_, 2.5; NaHCO_3_, 25.0; EDTA, 0.026; pH 7.4 at 37 °C and gassed with 95% O_2_ plus 5% CO_2_. Before contraction, a tension of 2.0 g was given to the aorta ring for 90 minutes. During this period, the Kreb’s solution was changed every 15 min. After the equilibration, aortic rings were challenged with 60 mM KCl. After washing and another 30 minutes equilibration period, contractile response was elicited by PE (1 μM). At the plateau of contraction, accumulative Ach or SNP was added into the organ bath to induce endothelium-dependent or -independent relaxation.

### Induction of atherosclerosis and analysis

Atherosclerotic model in rats were established by feeding high-fat diets, injecting vitamin D3, and inducing balloon. High-fat diets included 81.5% basic diets, 10% lard, 0.5% sodium cholate, 3% cholesterol, and 5% sugar. The dose of high-fat diets was 150 g/day. Common carotid arterial intima injury was induced after the rats were fed for 4 weeks. The rats were continuously fed with high-fat diets for 8 weeks. For analysis of atherosclerotic lesion, the common carotid artery was obtained and embedded in paraffin. Four consecutive sections (5 μm thickness) were collected from each rat and stained with Oil Red O for neutral lipids, and counterstained with hematoxylin to visualize the nuclei. Plaques were captured under the Olympus microscope connected to a QImaging Retiga CCD camera. The lesion size of each animal was obtained by the averaging of lesion areas in four sections from the same mouse.

### Immunohistochemistry

The carotid was fixed in 4% paraformaldehyde overnight, and then processed, embedded in paraffin, and sectioned at 4 μm. The deparaffinized, rehydrated were microwaved in citrate buffer for antigen retrieval. Sections were incubated in endogenous peroxidase (DAKO) and protein block buffer, and then with primary antibodies indicated overnight at 4 °C. Slides were rinsed with washing buffer and incubated with labelled polymer-horseradish peroxidase-antimouse/antirabbit antibodies followed by DAB + chromogen detection (DAKO). After final washes, sections were counterstained with hematoxylin. All positive staining was confirmed by ensuring that no staining occurred under the same conditions with the use of non-immune rabbit or mouse control IgG.

### Measurements of MDA, NO, cytokines, SOD activity, total cholesterol (TC), triglyceride (TG), low density lipoprotein (LDL), high density lipoprotein (HDL), glucose, apolipoprotein A-1 (ApoA-1), and apolipoprotein B (ApoB)

The commercial kits purchased from Nanjing Jiancheng Biological Company were used to measure the levels of MDA, SOD activity, NO, IL-1, IL-6, ICAM-1, VCAM-1, NF-kB, TC, TG, LDL, HDL, Glucose, ApoA-1, and ApoB in samples of aortic tissue or blood by following the recommended protocols.

### Statistical analysis

Data are reported as mean ± SEM. All data were analyzed with the use of 1 or 2-way ANOVA followed by multiple t-tests, and *P* < 0.05 were considered statistically significant.

## Results

### XMJ upregulates NO-related signaling in endothelial cells

**Figure 1:**
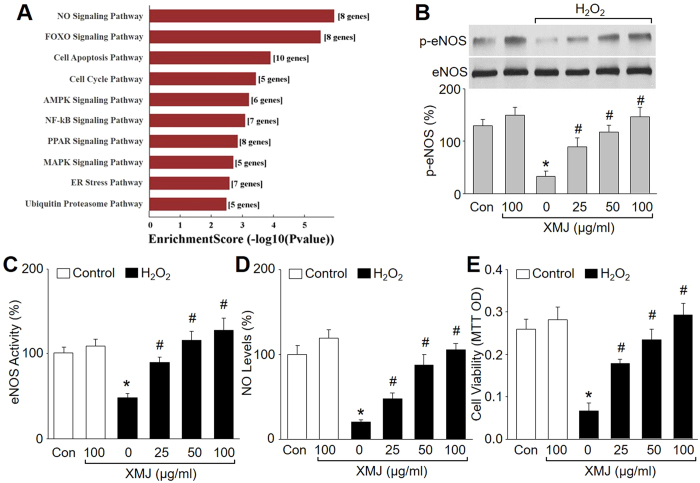
XMJ increases eNOS phosphorylation and activity, enhances NO generation, and improves cell viabilities in HUVECs. HUVECs were pretreated with XMJ (25, 50, 100 μg/ml) for 30 minutes and then incubated with H_2_O_2_ (0.2 mM) for 2 hours. (**A**) Differentially expressed genes were analyzed by mRNA array in cells treated with vehicle and XMJ (50 μg/ml). Signaling pathway analysis was performed by mapping genes to KEGG pathways. The p-value denotes the significance of the Pathway correlated to the conditions. (**B**) The eNOS phosphorylation was assayed by western blot in total cell lysates. The representative blots were cropped and the gels were run under the same experimental conditions. (**C**) The eNOS activity was determine by H^3^-L-arginine-based method. (**D**) Intracellular NO levels were detected by DAF fluorescence. (**E**) Cell viability was measured by MTT. All data were expressed as mean ± SEM. N is 3 in each group. *P < 0.05 *VS* Control, ^#^*P* < 0.05 *VS* H_2_O_2_ alone.

We firstly performed the mRNA array to scan which signaling pathways were altered by XMJ in endothelial cells. Thus, HUVECs were treated with XMJ and differentially expressed genes were analyzed. Signaling pathway analysis was performed by mapping genes to KEGG pathways. As shown in Fig. 1A, XMJ treatment dramatically affected signaling pathways related to NO, FOXO, apoptosis, cell cycle, AMPK, MAPK, NF-kB, PPAR, etc. Among these pathways, NO signaling was in the pecking order. These results demonstrate that NO signaling is a main target of XMJ.

### XMJ increases eNOS phosphorylation and activity in endothelial cells

We have previously reported that coupled eNOS is a key for NO production^16^. To determine whether XMJ recouples eNOS, we investigated the effects of XMJ in eNOS Ser1177 phosphorylation, which represents an active eNOS form in endothelial cells^17^. As shown in Fig. 1B, treatment of HUVECs with H_2_O_2_ decreased eNOS phosphorylation. However, preincubation of HUVECs with XMJ (25, 50, 100 μg/ml) increased eNOS phosphorylation in H_2_O_2_-treated cells. Consistent with eNOS phosphorylation, XMJ also concentration-dependently increased eNOS activity in H_2_O_2_-treated HUVECs (Fig. 1C). These data indicate that XMJ activates eNOS in endothelial cells.

### XMJ improves endothelial cell functions in H_2_O_2_-treated endothelial cells

The important function of endothelial cell is to generate eNOS-derived NO to regulate vascular tone^18^. We then investigated whether XMJ affected cell functions by determining NO productions and cell viabilities in H_2_O_2_-treated HUVECs. As depicted in Fig. 1D and E, incubation of HUVECs with H_2_O_2_ dramatically reduced NO productions and damaged cell viabilities. However, XMJ concentration-dependently reversed NO generations and cell proliferations, consistent with our previous report^19^.

### XMJ reduces H_2_O_2_-induced oxidative stress in endothelial cells

**Figure 2:**
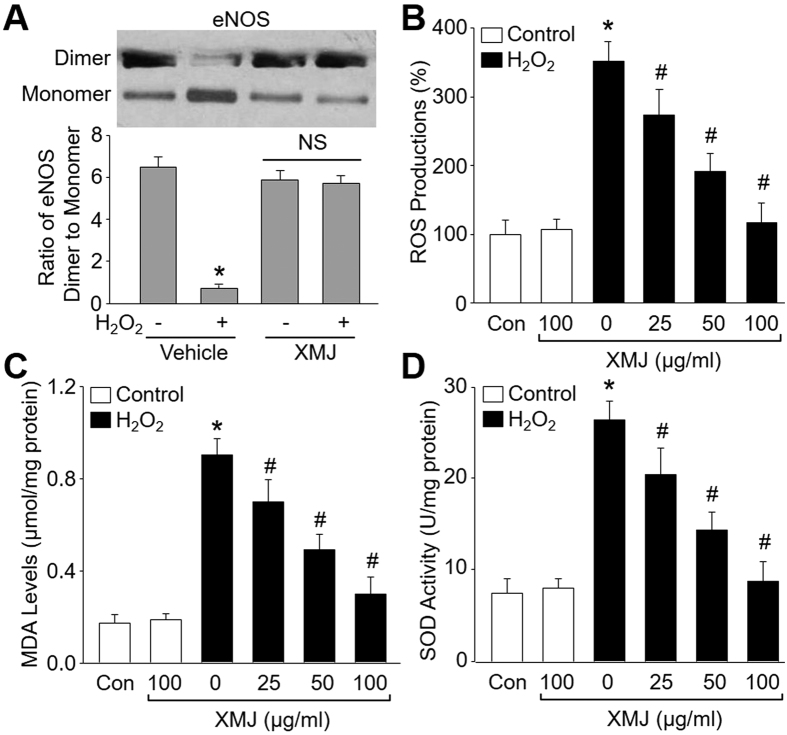
XMJ reduces oxidative stress in HUVECs treated with H_2_O_2_. HUVECs were pretreated with XMJ (25, 50, 100 μg/ml) for 30 minutes and then incubated with H_2_O_2_ (0.2 mM) for 2 hours. (**A**) Distributions of eNOS dimer and monomer were determined by cold western blot analysis. (**B**) ROS productions were detected by DHE fluorescence. (**C**) MDA levels in total cell lysates. (**D**) SOD activity in total cell lysates. All data were expressed as mean ± SEM. N is 3 in each group. *P < 0.05 *VS* Control, ^#^*P* < 0.05 *VS* H_2_O_2_ alone. NS indicates no significance.

The formation of eNOS dimer is essential for eNOS function to produce NO^20^. We then examined the effects of XMJ on eNOS dimer distribution and oxidative stress, which is a consequence of eNOS uncoupling^12^. As indicated in Fig. 2A, H_2_O_2_ reduced the distribution of eNOS dimer, which was reversed by XMJ. Further, incubation of HUVECs with H_2_O_2_ remarkably increased ROS productions (Fig. 2B) and the content of MDA (Fig. 2C) in cultured endothelial cells, which is formed when ROS reacts with polyunsaturated fatty acid chain in membrane lipids. Besides, H_2_O_2_ remarkably increased the secretions of cytokines, including ICAM-1, VCAM-1, IL-1, IL-6, and NF-κB from HUVECs (Table 1). However, preincubation of HUVECs with XMJ inhibited the enhancements of ROS productions, MDA content, and secretions of cytokines induced by H_2_O_2_ in dose-dependent manner, demonstrating that XMJ suppresses oxidative stress and inflammation in endothelial cells.

The effects of XMJ on oxidative stress were further confirmed by determining the activity of SOD, which is an important anti-oxidative enzyme to catalyze the dismutation of ROS. Conversely, H_2_O_2_ reduced SOD activity (Fig. 2D), which was abolished by pretreatment of cells with XMJ.

### XMJ increases BH4 levels in H_2_O_2_-treated HUVECs

**Figure 3:**
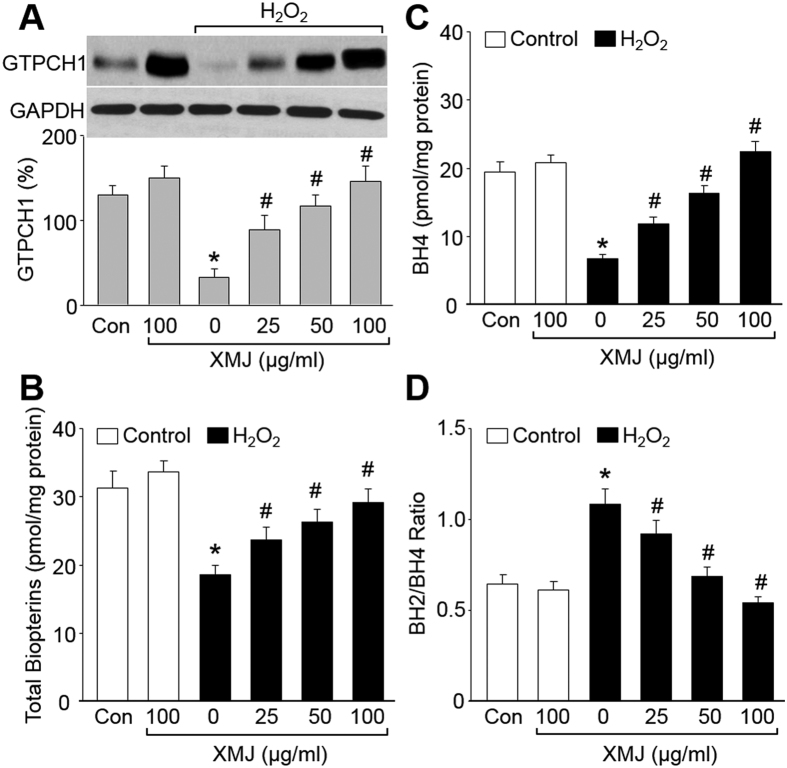
XMJ increases BH4 levels in H_2_O_2_-treated HUVECs. HUVECs were pretreated with XMJ (25, 50, 100 μg/ml) for 30 minutes and then incubated with H_2_O_2_ (0.2 mM) for 2 hours. (**A**) GTPCH1 protein level was measured by western blot in total cell lysates. The representative blots were cropped and the gels were run under the same experimental conditions. (**B** and **C**) The levels of total biopterins in (**B)** and BH4 in (**C)** were determined by HPLC. (**D**) BH2/BH4 ratio was calculated. All data were expressed as mean ± SEM. N is 3 in each group. *P < 0.05 *VS* Control, ^#^*P* < 0.05 *VS* H_2_O_2_ alone.

The function of eNOS is to produce NO, which is determined by BH4^21^. Intracellular BH4 levels are dictated by a balance of *de novo* synthesis, BH4 oxidation to BH2^22^. *De novo* biosynthesis of BH4 is regulated by GTP cyclohydrolase 1 (GTPCH1), a homodecameric protein consisting of 25 kDa subunits in mammalian cells^23^. Thus, we examined the effects of XMJ on GTPCH1 protein levels by western blot. As shown in Fig. 3A–C, XMJ dose-dependently increased the levels of GCH1 protein, total biopterins, and BH4 in H_2_O_2_-treated HUVECs, revealing that XMJ increased BH4 level through *De novo* biosynthesis.

Astaxanthin, as a component of XMJ, is an antioxidant^24^. We speculated that XMJ increased BH4 level through inhibition of BH4 oxidation. To testify this notion, we calculated the BH2/BH4 ratio (Fig. 3D). H_2_O_2_, as an oxidant, increased the ratio of BH2 to BH4. However, XMJ reversed the ration of BH2 to BH4, suggesting that XMJ increased BH4 level through inhibition of BH4 oxidized to BH2.

### Inhibition of eNOS attenuates XMJ-improved endothelial cell functions in H_2_O_2_-treated endothelial cells

**Figure 4:**
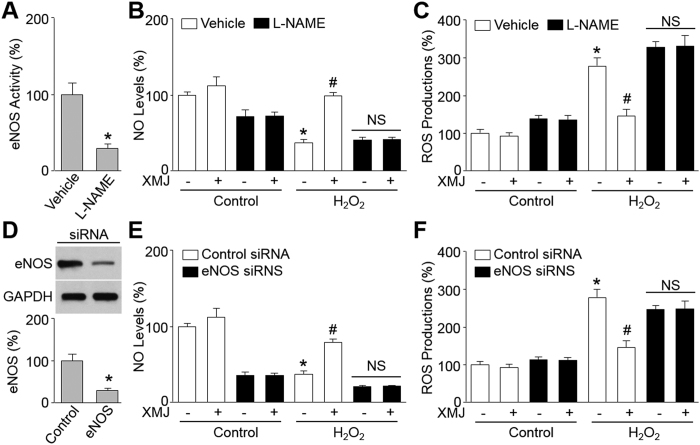
Inhibition of eNOS abolished the beneficial effects of XMJ in HUVECs. (**A**–**C**) HUVECs were pretreated with XMJ (50 μg/ml) plus L-NAME (1 mM) for 30 minutes and then incubated with H_2_O_2_ (0.2 mM) for 2 hours. eNOS activity was determined by H^3^-L-arginine-based method in (**A)**. Intracellular NO levels were detected by DAF fluorescence in (**B)**. ROS productions were detected by DHE fluorescence in (**C**). All data were expressed as mean ± SEM. N is 3 in each group. *P < 0.05 *VS* Vehicle or Control, ^#^*P* < 0.05 *VS* H_2_O_2_ alone. NS indicates no significance. (**D**–**F**) HUVECs were transfected with control or eNOS siRNA for 48 hours followed by treated with XMJ (50 μg/ml) in presence or absence of H_2_O_2_ (0.2 mM) for 2 hours. The levels of eNOS protein in (**D)**, intracellular NO in (**E)**, and ROS productions in (**F)** were determined, respectively. All data were expressed as mean ± SEM. N is 3 in each group. *P < 0.05 *VS* Control siRNA alone, ^#^*P* < 0.05 *VS* control siRNA plus H_2_O_2_. NS indicates no significance.

The role of eNOS recoupling in XMJ-induced protective effects in HUVECs was determined by inhibition of eNOS using L-NAME, as a well-known eNOS inhibitor^12^. As shown in Fig. 4A, L-NAME dramatically inhibited eNOS activity, compared to vehicle-treated cells. Preincubation of HUVECs with L-NAME (1 mM) bypassed XMJ-induced NO generations (Fig. 4B) and ROS reductions (Fig. 4C) in H_2_O_2_-treated HUVECs, demonstrating that XMJ improves endothelial cell functions, which depends on eNOS recoupling.

To exclude the possibility that these results were due to non-specific effects of L-NAME treatment, we repeated these experiments with HUVECs transfected with control siRNA or eNOS siRNA (Fig. 4D). As shown in Fig. 4E and F, genetic silence of eNOS mimicked the effects of L-NAME on XMJ-induced eNOS recoupling in HUVECs, compared to HUVECs transfected with control siRNA, implying that eNOS is required for XMJ to protect endothelial cells.

### XMJ recouples eNOS in isolated rat aortic rings *ex vivo*

**Figure 5:**
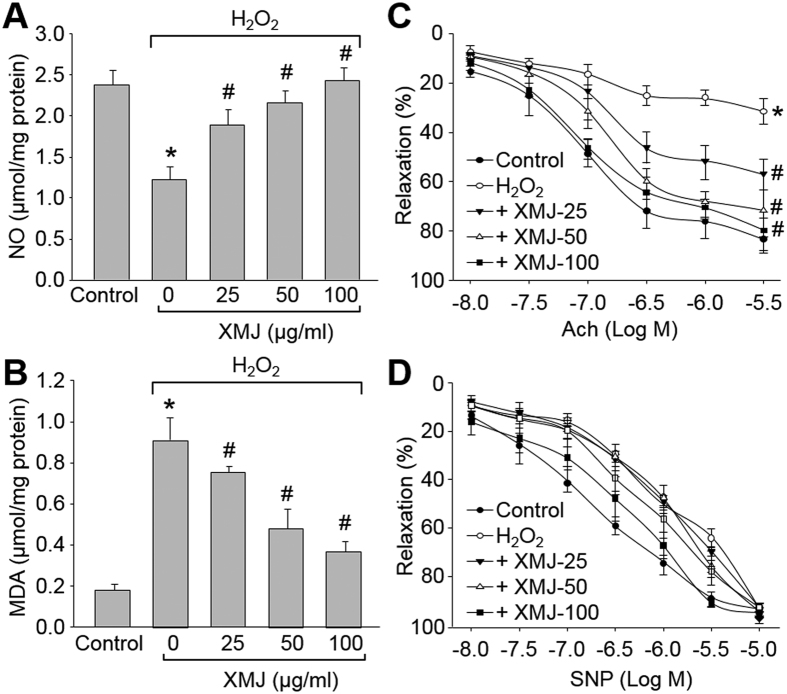
XMJ prevents endothelial dysfunction in aortic arteries isolated from rats *ex vivo*. The isolated rat aortic rings were incubated with XMJ (25, 50, 100 μg/ml) for 30 minutes followed by exposure to H_2_O_2_ (0.2 mM) for 6 hours. (**A**) NO levels and (**B**) MDA levels in aortic tissues were assessed, respectively. (**C**) The endothelium-dependent relaxation induced by acetylcholine (Ach) and (**D**) endothelium-independent relaxation elicited by sodium nitroprusside (SNP) were assayed in organ chamber. All data were expressed as mean ± SEM. N is 5 in each group. *P < 0.05 *VS* Control, ^#^*P* < 0.05 *VS* H_2_O_2_ alone.

Deficiency of eNOS-derived NO, as a major component to endothelium-dependent relaxation factor, is an early marker for endothelial dysfunction in atherosclerosis^14^,^25^. Thus, we performed *ex vivo* experiments to test whether XMJ recouples eNOS in isolated rat aortic rings by measuring NO and MDA levels. In Fig. 5A and B, H_2_O_2_ dramatically decreased NO content and increased MDA level. Pretreatment of aortic rings with XMJ concentration-dependently abolished H_2_O_2_-induced the abnormalities, suggesting that XMJ recouples eNOS *ex vivo*.

### XMJ preserves endothelium-dependent relaxation *ex vivo*

We then tested whether XMJ protects vascular endothelial functions by incubating rat aortic rings with H_2_O_2_ *ex vivo*. As shown in Fig. 5C, exposure of aortic rings to H_2_O_2_ dramatically impaired Ach-induced endothelium-dependent relaxation, which was concentration-dependently reversed by XMJ. However, SNP-induced endothelium-independent relaxation was not altered in all groups (Fig. 5D). Collectively, these data indicate that XMJ functions as a protector on vascular endothelium.

### Administration of XMJ reduces the size of carotid atherosclerotic plaque in rats fed with high fat diet *in vivo*

We finally performed *in vivo* experiments to investigate whether XMJ produces beneficial effects on atherosclerosis. To this end, rats were intragastrically gavaged with XMJ (0.6, 2.0, 6.0 g/kg/day) for 4 weeks followed by high fat diet for another 4 weeks. Vascular endothelium in carotid was damaged by performing surgery of balloon injury. Lovastatin and another Chinese medicine ZBT served as positive controls. As shown in Table 2, XMJ, lovastatin, and ZBT lowered the levels of TC, TG, LDL, but elevated the levels of HDL, ApoA-1, ApoB, without alteration of blood glucose.

**Figure 6:**
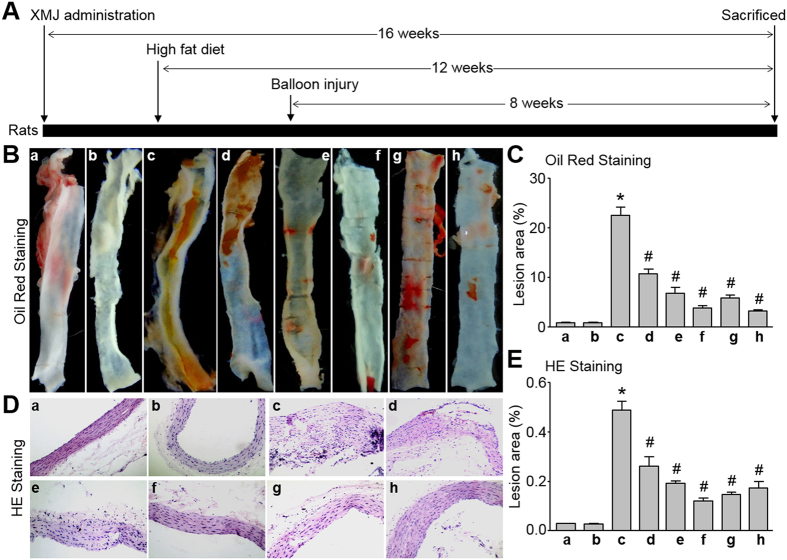
XMJ reduces the formation of carotid atherosclerotic plaque in rats. (**A**) The protocol of animal experiment. (**B** and **C**) At the end of experiment, carotid artery was subjected to perform morphological analysis by Oil Red staining in (**B)** and quantitative analysis of atherosclerotic plaque size in (**C)**. (**D** and **E**) Histological analysis by HE staining in (**D**) and quantitative analysis of atherosclerotic plaque size in (**E**) were performed. a, Sham; b, Sham + XMJ-6.0; c, Atherosclerosis (AS); d, AS + XMJ-0.6; e, AS + XMJ-2.0; f, AS + XMJ-6.0; g, Lovastatin; h, ZBT. All data were expressed as mean ± SEM. 10–15 rats per group. *P < 0.05 *VS* Control, ^#^*P* < 0.05 *VS* AS alone.

The atherosclerotic plaque was determined by Oil Red staining and HE staining. As indicated in Fig. 6B–E, conduction of balloon injury plus high fat diet in rats markedly induced the formation of atherosclerotic plaque in carotid arteries, compared to rats from control and sham groups. Importantly, XMJ administration dramatically reduced the size of atherosclerotic plaque in carotid arteries, which was in dose-dependent manner. Interestingly, the effects of XMJ at 6.0 g/kg/day on atherosclerotic plaque size were more obvious than lovastatin and ZBT at conventional dose. These data indicate that XMJ prevents the growth of atherosclerotic plaque *in vivo*.

### XMJ recouples eNOS in rats *in vivo*

**Figure 7:**
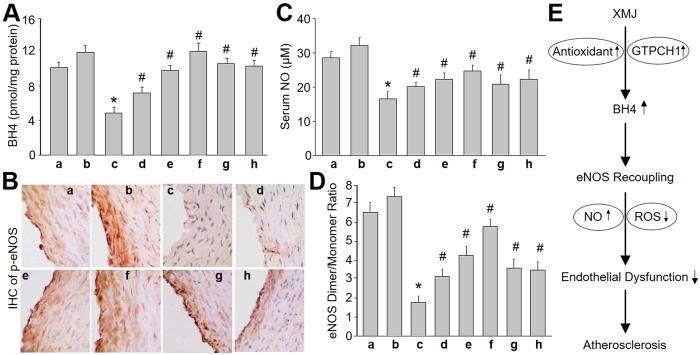
XMJ recouples eNOS and inhibits oxidative stress in atherosclerotic rats. The protocol of animal experiment was described in Fig. 6A. Carotid artery was subjected to assess the levels of (**A**) BH4 by HPLC and (**B**) p-eNOS by IHC. Blood was collected to assay (**C**) serum NO level by Griess method. (**D**) Distributions of eNOS dimer and monomer were determined by cold western blot analysis. a, Sham; b, Sham + XMJ-6.0; c, Atherosclerosis (AS); d, AS + XMJ-0.6; e, AS + XMJ-2.0; f, AS + XMJ-6.0; g, Lovastatin; h, ZBT. All data were expressed as mean ± SEM. 10–15 rats per group. *P < 0.05 *VS* Control, ^#^*P* < 0.05 *VS* AS alone. (**E**) Proposed mechanism of XMJ against atherosclerosis.

To illustrate whether eNOS recoupling is involved in the anti-atherosclerotic effects of XMJ *in vivo*, we examined the function of eNOS by determining the redox state in atherosclerotic rats. Expectedly, administration of XMJ, as well as lovastatin and ZBT, reserved BH4 levels (Fig. 7A) and eNOS phosphorylations in carotid artery (Fig. 7B), and serum NO level (Fig. 7C) in rats with atherosclerosis. Analysis of eNOS dimer/monomer distribution also indicated that XMJ rescued the normal levels of eNOS dimer in carotid artery from rats (Fig. 7D). Further, XMJ, lovastatin and ZBT, increased serum SOD activity and decreased serum levels of MAD, but reduced serum levels of cytokines, including ICAM-1, VCAM-1, IL-1 and IL-6 (Table 3) in atherosclerotic rats. In sum, it indicates that XMJ may increase BH4 through GTPCH1-dependent *de novo* biosynthesis and inhibition of BH4 oxidation, resulting in eNOS recoupling by increasing NO generation and decreasing ROS productions in endothelial cells (Fig. 7E).

## Discussions

The major finding of the present project is that XMJ prevents atherosclerosis in rats. Previous studies from us have shown that XMJ alleviates cardiovascular and cerebrovascular diseases and decreases blood lipid levels in rats. In cell studies, we also reported that XMJ has clear anti-inflammatory and antioxidant effects and significantly inhibits the proliferation and migration of VSMCs. These observations reveal the potential effects of XMJ against atherosclerosis. This was confirmed by this study that XMJ dose-dependently prevents the formation of carotid atherosclerotic plaque induced by balloon injury plus high fat diet in rats. Further, we compared the effects of XMJ on atherosclerosis to lovastatin and ZBT, both widely used in clinical patients to prevent atherosclerosis. We found the effects of XMJ at medium to high dose on reducing atherosclerotic plaque size were stranger than lovastatin and ZBT in conventional doses, indicating the broad applications of XMJ clinically.

By using gene expression array, we identified several signaling pathways related to NO, FOXO, apoptosis, cell cycle, AMPK, MAPK, NF-kB, and PPAR, which were affected by XMJ in endothelial cells. We choose NO signaling as a candidate because NO signaling was in the pecking order among these signaling, consistent with our previous studies that XMJ produces the anti-inflammatory and antioxidant effects in HUVECs by increasing NO productions^19^,^26^,^27^. This is also highly related to clinical investigations that Chinese medicine has globally recognized to improve health conditions by activating NO signaling^28^.

Mechanically, we uncovered that the anti-atherosclerotic action of XMJ is mediated by BH4-dependent eNOS recoupling. eNOS uncoupling has been implicated in a number of vascular diseases, such as hypertension^7^, atherosclerosis^8^, and diabetes^29^, and is often accompanied by a reduction in BH4 levels. In this study, we observed that XMJ increased the intracellular BH4 levels through two pathways. One is to increase *de novo* synthesis of BH4 from GTPCH1 by upregulating the protein level. Another one is to inhibit BH4 oxidation to BH2. Previously, we have reported that XMJ activates NO-cGMP signaling to protect functions of HUVECs. Our results further demonstrate that normalization of BH4-dependent eNOS recoupling by XMJ contributes to maintain cell viability in endothelial cells. In addition, BH4 supplementation in vessel rings from animals with atherosclerosis, diabetes, or hypertension reduces endothelial dysfunction^9^,^10^,^11^. BH4 administration augments NO-mediated vasodilation in diabetic patients^30^ and smokers^31^. GTPCH1 gene transfer reverses BH4 deficiency and restores eNOS function in both endothelial cells and vessels isolated from diabetic rats^32^ and rats with low-renin hypertension^33^. From our investigations, treatment of XMJ should be considered as an alternative method to replace BH4 supplementation or GTPCH1 gene transfer to protect endothelial functions.

In summary, the present study supports a novel function of XMJ which recouples eNOS to improve endothelial dysfunction. In this way, XMJ inhibits the growth of atherosclerotic plaque in rat carotid artery. The finding that XMJ attenuates endothelial dysfunction may have broad applications since endothelial dysfunction is a common character at the beginning and in the progress in a number of vascular diseases including atherosclerosis^34^,^35^, hypertension and diabetes^36^.

## Additional Information

**How to cite this article**: Yin, Y.-L. *et al*. Traditional Chinese medicine xin-mai-jia recouples endothelial nitric oxide synthase to prevent atherosclerosis *in vivo. Sci. Rep.* **7**, 43508; doi: 10.1038/srep43508 (2017).

**Publisher's note:** Springer Nature remains neutral with regard to jurisdictional claims in published maps and institutional affiliations.

## Figures and Tables

**Table 1:** Secretion of cytokines from cultured HUVECs.

Groups	ICAM-1 (ng/L)	VCAM-1 (μg/L)	IL-1 (ng/L)	IL-6 (ng/L)	NF-κB (ng/L)
Control	28.91 ± 3.65	36.49 ± 1.22	14.23 ± 1.25	5.95 ± 0.45	63.33 ± 8.98
Sham	36.33 ± 4.39	38.78 ± 2.39	13.93 ± 1.36	6.51 ± 0.65	77.97 ± 9.65
Atherosclerosis	71.18 ± 6.67^a^	44.81 ± 2.09	18.34 ± 2.14	7.24 ± 0.92	200.46 ± 25.68^a^
AS + XMJ-0.6	62.78 ± 5.47	38.94 ± 2.47	15.49 ± 2.31	6.48 ± 0.77	86.09 ± 8.64^b^
AS + XMJ-2.0	36.33 ± 7.32^b^	36.66 ± 1.58	14.75 ± 2.04	6.05 ± 0.84	67.43 ± 7.81^b^
AS + XMJ-6.0	39.29 ± 4.57^b^	43.38 ± 2.39	16.07 ± 2.55	7.21 ± 0.98	65.84 ± 10.32^b^
Lovastatin	38.55 ± 4.16^b^	35.48 ± 1.96	14.63 ± 1.57	6.05 ± 0.78	70.79 ± 8.54^b^
Zhibituo	34.10 ± 4.12^b^	36.49 ± 2.68	14.79 ± 1.69	6.19 ± 0.65	70.51 ± 7.69^b^

N is 3 in each group. Data were expressed by mean ± SEM.

**Table 2:** Serum lipids and glucose levels in rats.

Groups	TC (μM)	TG (μM)	LDL (μM)	HDL (μM)	ApoA-1 (μM)	ApoB (μM)	GLU (mM)
Control	0.72 ± 0.09	1.13 ± 0.09	0.13 ± 0.03	0.42 ± 0.08	0.17 ± 0.04	0.13 ± 0.03	4.17 ± 0.12
Sham	0.54 ± 0.07	0.42 ± 0.04	0.11 ± 0.02	0.47 ± 0.07	0.18 ± 0.05	0.11 ± 0.02	5.01 ± 0.17
Atherosclerosis	1.13 ± 0.16^a^	1.82 ± 0.12^a^	0.28 ± 0.05^a^	0.27 ± 0.05^a^	0.07 ± 0.01^a^	0.02 ± 0.01^a^	5.30 ± 0.26
AS + XMJ-0.6	0.48 ± 0.06^b^	0.97 ± 0.07^b^	0.19 ± 0.05^b^	0.39 ± 0.06^b^	0.14 ± 0.03^b^	0.05 ± 0.01^b^	4.83 ± 0.21
AS + XMJ-2.0	0.44 ± 0.06^b^	0.84 ± 0.06^b^	0.15 ± 0.03^b^	0.41 ± 0.07^b^	0.09 ± 0.01	0.09 ± 0.02^b^	4.44 ± 0.18
AS + XMJ-6.0	0.55 ± 0.07^b^	0.99 ± 0.12^b^	0.16 ± 0.02^b^	0.32 ± 0.04	0.11 ± 0.02	0.11 ± 0.03^b^	4.05 ± 0.17
Lovastatin	0.51 ± 0.06^b^	0.48 ± 0.07^b,c^	0.22 ± 0.04^b^	0.33 ± 0.06	0.08 ± 0.01	0.08 ± 0.01^b^	4.51 ± 0.19
Zhibituo	0.55 ± 0.08^b^	0.58 ± 0.08^b,c^	0.23 ± 0.03^b^	0.35 ± 0.06	0.09 ± 0.02	0.06 ± 0.01^b^	4.95 ± 0.28

AS, Atherosclerosis; TC, total cholesterol; TG, triglycerides; LDL, low-density lipoprotein cholesterol; HDL, high-density lipoprotein cholesterol; GLU; glucose. N is 12 in each group. Data were expressed by mean ± SEM.

**Table 3:** Serum levels of cytokines, SOD activity, and MDA in rats.

Groups	ICAM-1 (nM)	VCAM-1 (μM)	IL-1 (nM)	IL-6 (nM)	MDA (μM)	SOD (kU/L)
Control	283.33 ± 33.64	38.46 ± 3.23	132.25 ± 16.25	56.93 ± 4.45	1.31 ± 0.23	56.54 ± 4.51
Sham	357.35 ± 34.49	36.76 ± 4.32	141.94 ± 17.46	67.54 ± 4.64	1.26 ± 0.18	55.98 ± 5.31
Atherosclerosis	789.76 ± 65.53^a^	46.84 ± 2.04	176.54 ± 26.54^a^	76.54 ± 6.97	4.38 ± 0.33^a^	17.65 ± 2.19^a^
AS + XMJ-0.6	678.65 ± 56.66	37.54 ± 4.42	154.45 ± 25.35	69.54 ± 6.76	3.29 ± 0.47	22.08 ± 3.27
AS + XMJ-2.0	377.65 ± 78.65^b^	38.66 ± 3.55	146.65 ± 24.44^b^	69.09 ± 4.86	2.54 ± 0.41^b^	38.47 ± 3.31^b^
AS + XMJ-6.0	399.76 ± 43.32^b^	44.33 ± 4.33	165.08 ± 23.53	72.23 ± 8.96	2.47 ± 0.39^b^	44.47 ± 5.63^b^
Lovastatin	422.25 ± 45.21^b,c^	37.83 ± 3.93	154.65 ± 14.56	65.56 ± 8.74	3.69 ± 0.33	21.41 ± 2.39^b,c^
Zhibituo	354.35 ± 44.32^b,c^	36.59 ± 3.65	155.49 ± 14.65	67.79 ± 6.67	3.54 ± 0.26	26.59 ± 2.37

AS, Atherosclerosis; N is 12 in each group. Data were expressed by mean ± SEM.
